# Posterior Reversible Encephalopathy Syndrome in a Pediatric Intensive Care Unit: A Case Series

**DOI:** 10.7759/cureus.50658

**Published:** 2023-12-17

**Authors:** Beatriz Teixeira, Vera Gonçalves, Ana Lúcia Cardoso, Sofia Ribeiro Fernandes, Liliana Rocha, Cristina Garrido, Alzira Sarmento

**Affiliations:** 1 Paediatric Department, Centro Materno Infantil do Norte, Centro Hospitalar Universitário de Santo António, Porto, PRT; 2 Paediatric Department, Unidade Local de Saúde do Alto Minho, Viana do Castelo, PRT; 3 Paediatric Intensive Care Department, Centro Materno Infantil do Norte, Centro Hospitalar Universitário de Santo António, Porto, PRT; 4 Paediatric Nephrology, Centro Materno Infantil do Norte, Centro Hospitalar Universitário de Santo António, Porto, PRT; 5 Paediatric Neurology Department, Centro Materno Infantil do Norte, Centro Hospitalar Universitário de Santo António, Porto, PRT

**Keywords:** antihypertensive therapy, seizure, vasogenic edema, hypertension, posterior reversible encephalopathy syndrome

## Abstract

Posterior reversible encephalopathy syndrome (PRES) is a reversible clinical-radiographic abnormality. It is characterized by headache, altered consciousness, seizures, and visual disruption, in addition to characteristic white matter edema lesions in the parieto-occipital areas of the brain. Early detection and treatment are crucial to prevent irreversible damage. This paper presents the cases of three patients with PRES with concurrent diagnoses of glomerulonephritis, Guillain-Barré syndrome, and sickle cell disease. All patients experienced systemic hypertension, seizures, and altered consciousness. All patients were admitted to intensive care for decreased level of awareness or *status epilepticus* requiring invasive mechanical ventilation. Anticonvulsants and antihypertensive therapy were essential. No chronic complications were recorded.

## Introduction

Posterior reversible encephalopathy syndrome (PRES) is a clinical-radiographic syndrome of heterogeneous etiologies first described by Hinchey et al. in 1996. It was initially defined as a reversible neurological condition characterized by headache, altered mental function, seizures, and vision loss, associated with neuroimaging findings suggesting white matter edema, mainly in the posterior parietal-occipital regions of the brain [1]. Cumulating evidence has revealed that PRES is not always fully reversible and that abnormalities are not exclusive of parieto-occipital lobes white matter [2]. Therefore, PRES has revealed further presentations than initially recognized [3].

Female adolescents appear to have a higher incidence of PRES [4]. Incidence in children remains uncertain. According to some studies, PRES-related hospitalizations for the pediatric population occur at a rate of 0.04% [4], with a rate of 0.4% in pediatric intensive care units (PICUs) [5].

Several disorders and risk factors have been associated with PRES, such as hypertension (HTN), immunosuppressive therapy, renal diseases, and autoimmune disorders. The pathogenesis of PRES has yet to be entirely understood. Several mechanisms have been suggested, all with a common result: cerebral vasogenic edema (VE) [6]. Four theories have been proposed to explain PRES. The "vasogenic theory" postulates that elevated blood pressure (BP) may exceed cerebral autoregulation mechanisms, leading to arterioles dilation and extravasation of plasma into the brain parenchyma. The “cytotoxic theory” claims that toxins and drugs may cause endothelial damage, leading to capillary leakage and VE. The “immunogenic theory” advances that T-cell activation and cytokine release result in endothelial dysfunction and disturbed autoregulatory response. The “neuropeptide theory” points out that the release of vasoconstrictors causes vasospasm with ischemia and VE. These mechanisms may coexist and contribute differently among patients [7].

PRES symptoms are nonspecific and develop over several hours to days. Seizures are the most common symptom, typically occurring at presentation, mostly as generalized tonic-clonic (TC) seizures [5]. Altered consciousness, headache, nausea, vomiting, and vision impairment are also common [7].

Brain magnetic resonance imaging (MRI) revealing VE lesions is essential for diagnosis. Bilateral hyperintensities on T2-weighted and fluid-attenuated inversion recovery (FLAIR) sequences are the typical findings. With treatment, these findings are expected to disappear within a few days to weeks [2,8].

Early recognition and approach of PRES are essential. PRES management is supportive, comprising the treatment of the underlying cause, removal of potentially causative drugs, and symptoms-directed management (such as HTN and seizure control) [9]. Prognosis is generally good, although life-threatening complications can occur [10], such as hemorrhage, infarction, and epilepsy. These complications are more frequent in children than initially thought [10,11].

By describing three patients diagnosed with PRES and admitted to a PICU, we aimed to discuss PRES's diagnostic and therapeutic approach.

## Case presentation

The clinical records of three patients admitted to our PICU with a discharge diagnosis of PRES were reviewed. The analyzed data included age, sex, underlying medical conditions, recent medication, clinical presentation, values of BP at admission, neuroimaging findings, therapeutic approach, length of PICU and hospital stay, and outcomes.

Case 1

An 11-year-old boy recently diagnosed with acute post-infectious glomerulonephritis, under oral prednisolone, was admitted to the pediatric emergency department (ED) for a generalized TC seizure. Severe headache, nausea, and vomiting two hours before the seizure were reported. On admission to the ED, the patient was lethargic, with a Glasgow coma scale (GCS) score of 9 (E2V2M5), and hypertensive, with systolic BP above the 95th percentile (128 mmHg), with no other relevant findings on physical examination. Head computed tomography (CT) scan and cerebrospinal fluid (CSF) analysis were unremarkable. A few minutes after the initial evaluation, he presented another generalized TC seizure that was unresponsive to antiepileptic treatment (diazepam, phenytoin, and sodium valproate), requiring midazolam infusion for seizure control. Subsequently, he was intubated and transferred to the PICU under mechanical ventilation.

**Figure 1:**
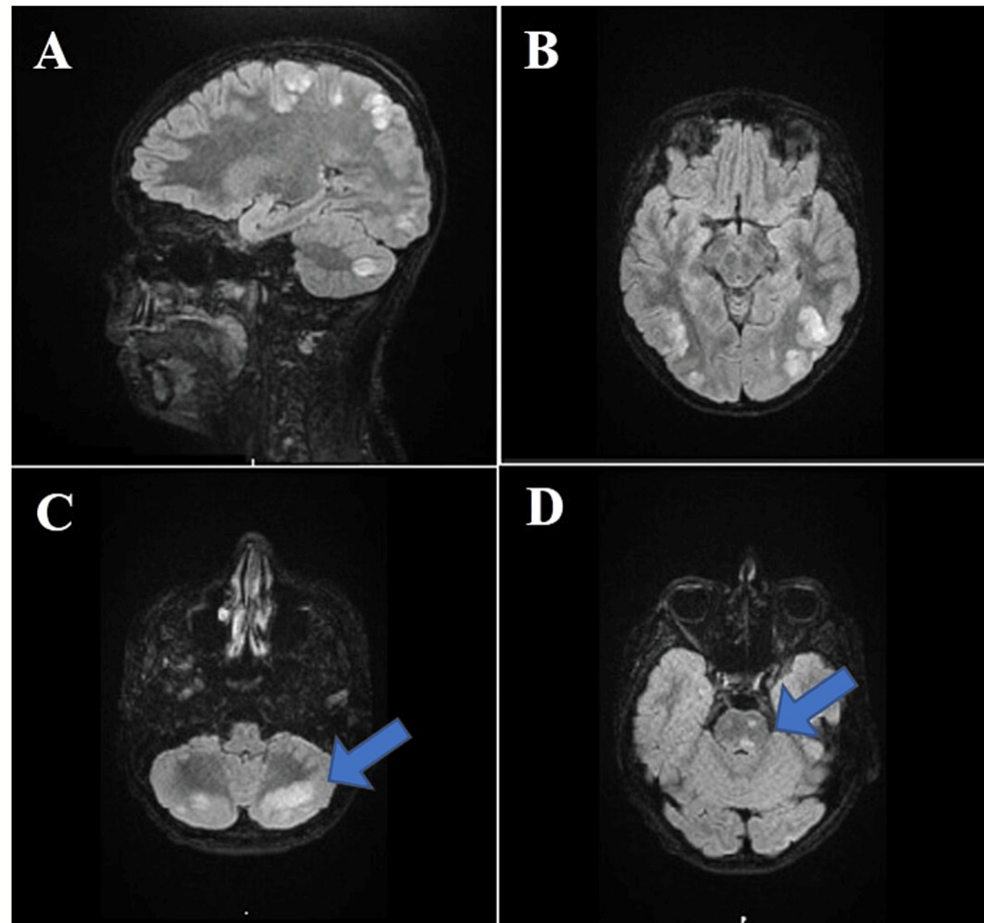
MRI findings consistent with PRES (patient 1). Multiple high-intensity lesions in cortical and subcortical white matter on the parieto-occipital and posterior frontal regions (images A and B), posterior area of cerebellar hemispheres (image C, arrow), and pontomesencephalic transition (image D, arrow).

On admission to the PICU, he presented with a persistently elevated systolic and diastolic BP (up to 135/94 mmHg), with a heart rate (HR) of 92 beats per minute (bpm) and isochoric pupils. An emergent MRI was performed, revealing symmetric high-intensity lesions in cortical and subcortical white matter on parieto-occipital and posterior frontal regions, posterior area of cerebellar hemispheres, and pontomesencephalic transition, on T2-weighted and FLAIR sequences (Figure 1). No water molecules diffusion restriction was detected on the diffusion-weighted imaging (DWI) sequence.

A diagnosis of PRES was established, and the patient was started on labetalol infusion and antiepileptic therapy with sodium valproate. Prednisolone was discontinued.

During the first two days after admission, there was a gradual drop in BP of around 20% per day. On day 3, midazolam was withdrawn, and the patient was successfully extubated. No additional seizures were recorded.

For suboptimal BP control, as well as the persistence of confusion and headache, the patient was started on amlodipine and enalapril, with complete resolution of neurological symptoms on day 4 and normalization of BP on day 10, allowing labetalol suspension. The patient was then shifted to the pediatric ward.

A comprehensive diagnostic approach to investigate an underlying cause of HTN and detect end-organ damage was performed. It included cardiac, abdominal, and renal ultrasounds, and laboratory studies (urea and creatinine, electrolytes, coagulation studies, C3, C4, and CH50, immunologic studies, and urinalysis). No remarkable findings were found except for an elevated antistreptolysin-O antibody titer.

Corticosteroids were restarted with strict BP control for hematuria recurrence and acute kidney injury. The patient was discharged home on day 23 under steroid therapy, sodium valproate, lisinopril, and amlodipine, with no neurological deficits. He was successfully weaned off corticosteroid and antihypertensive therapy within three months after discharge. He remained under sodium valproate for one year after the episode due to the finding of occipital focal paroxysmal activity on a follow-up electroencephalogram (EEG), without clinical seizures. Control MRI performed one year after PRES was normal. At 3.5 years of follow-up, the patient is neurologically asymptomatic and has presented with no further seizures.

Case 2

A three-year-old boy with no relevant medical history was admitted to ED for unsteady gait with ataxy and frequent falls, headache, and irritability. He had fever and respiratory symptoms a week prior to presentation. At physical examination, he presented with meningeal irritation signs without fever and BP between the 50th and 90th percentile. Ocular fundoscopy showed no papillary edema. Brain angio-CT and CSF analysis performed on admission showed no relevant changes. Encephalitis was suspected, and the patient was hospitalized and started on empirical antimicrobials (ceftriaxone, ciprofloxacin, and acyclovir).

On day 5, he was admitted to the PICU for clinical deterioration with progressive aphonia, perianal and lower limb pain, superior and inferior extremity weakness with absent deep tendon reflexes, and altered mental status, initially characterized by irritability and progressing to periods of confusion and somnolence. He also presented with a worsening hypertensive hemodynamic profile and had three generalized TC seizures treated with intravenous diazepam.

On PICU admission, the patient was tachycardic (HR 154 bpm), with BP above the 95th percentile (BP up to 139/107 mmHg). His mental state further deteriorated, progressing to coma and requiring intubation and mechanical ventilation. Since BP normalized after benzodiazepine and opiate infusions were started, no antihypertensive treatment was required at this moment.

**Figure 2:**
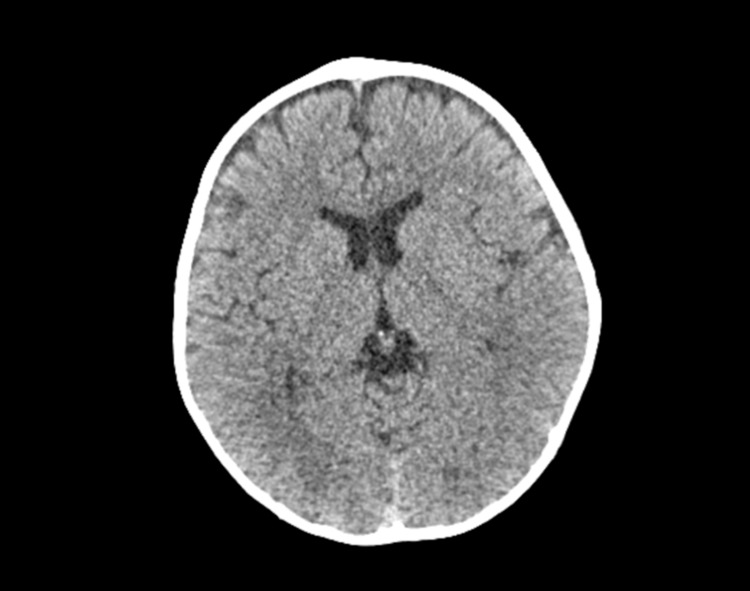
CT findings consistent with PRES (patient 2) Bilateral occipital hypodense white matter lesions on the parieto-occipital regions.

A repeat head CT scan revealed bilateral occipital hypodense white matter lesions suggestive of PRES (Figure 2). Repeat CSF analysis was acellular, with mild protein elevation and albuminocytologic dissociation. Immunologic work-up was positive for anti-GM1 antibody. He was then started on levetiracetam and intravenous immunoglobulin for a suspicion of Guillain-Barré syndrome (GBS) with dysautonomia complicated with PRES.

Electromyography confirmed a demyelinating sensory and motor polyneuropathy, affecting the upper and lower limbs, compatible with demyelinating GBS. The electroencephalogram revealed no paroxysmal activity. Extensive investigation into the causes of acute encephalitis and HTN as well as for the identification of end-organ damage did not reveal changes. It included cardiac, abdominal, and renal ultrasounds, and laboratory studies.

On day 3 after PICU admission, the patient was successfully extubated. After sedation withdrawal, he presented with a new rise in BP above the 95th percentile (up to 142/102 mmHg). Therefore, a labetalol infusion was started, which was later stopped on day 9, after normalization of BP.

**Figure 3:**
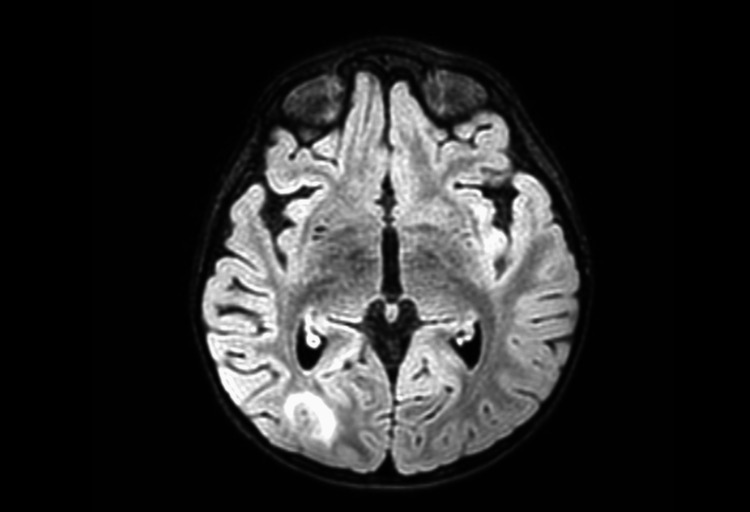
MRI findings consistent with PRES (patient 2) Parieto-occipital high-intensity lesions in the white matter.

On day 5 after PICU admission, a brain MRI was performed (Figure 3), which revealed enhancement of cauda equina nerve roots and parieto-occipital high-intensity lesions in white matter on T2-weighted and FLAIR sequences, with no water molecules diffusion restriction on DWI sequence.

On day 9, he was shifted to the ward due to an improvement in neurological deficits with no seizure recurrence. The patient was discharged home on day 21 with normal BP and without antiepileptic therapy. At 4-year follow-up, no recurrence of the symptoms was noted.

Case 3

A nine-year-old boy with sickle cell disease (SCD) was admitted to the pediatric ward for acute chest syndrome, for which he was under oxygen therapy, hydration, analgesia, and antimicrobial therapy (ceftriaxone plus azithromycin).

On day 8, he presented with a sudden deterioration of consciousness (GCS of 9, E2V2M5), with BP above the 95th percentile (up to 160/104 mmHg) and tachycardia (HR 154 bpm), and was admitted to the PICU.

**Figure 4:**
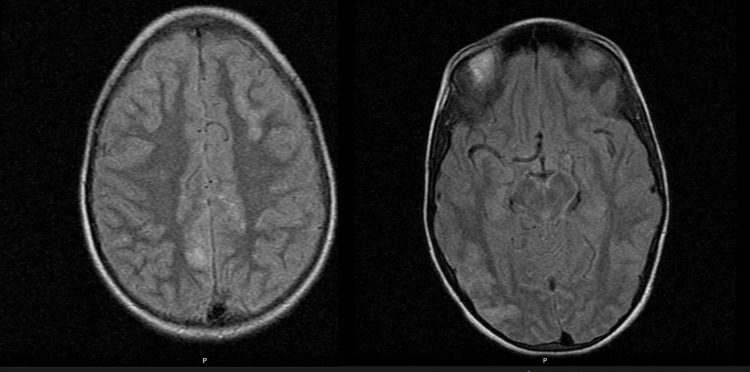
MRI findings consistent with PRES (patient 3) High-intensity cortical lesions located bilaterally on the posterior frontal regions and right-sided parieto-temporo-occipital regions.

On PICU admission, he had a generalized TC seizure, which was treated with diazepam and phenytoin, requiring intubation and mechanical ventilation due to a persistent coma. Brain CT scan was unremarkable, and brain MRI revealed findings consistent with PRES, with high-intensity cortical lesions located bilaterally on the posterior frontal regions and right-sided parieto-temporo-occipital regions on T2-weighted and FLAIR sequences (Figure 4); no water molecules diffusion restriction was detected on DWI sequence. The patient was started on amlodipine and nifedipine, with partial BP control.

The diagnostic investigation for an underlying cause of HTN or the identification of organ damage did not reveal any significant findings. It included echocardiogram, abdominal and renal echo-Doppler, and a range of laboratory studies including urea, creatinine, cystatin C, electrolytes, coagulation studies, renin, aldosterone, metanephrines, normetanephrines, complement, immunologic studies, and urinalysis.

The patient was extubated on day 10 with no neurological deficits or seizure recurrence. Enalapril was started for suboptimal BP control, with normalization on day 11, and he was then shifted to the ward.

He was discharged home on day 28 under enalapril, weaned off within two months, restarted seven months later due to HTN, and stopped after three years. At the five-year follow-up, he had no further seizures or neurological symptoms.

## Discussion

These cases serve to illustrate that PRES can manifest in association with different underlying diagnoses, shedding light on its diverse clinical presentations. According to the literature, kidney disorders are one of PRES's most important risk factors [4,5]. Concerning SCD, PRES is not a rare event, with a reported frequency of 10% in one study [12]. However, PRES can be underrecognized in SCD since the clinical distinction with stroke can be challenging [13]. Documented pediatric cases of PRES related to GBS or its variants are rare [14].

The three patients presented with seizures and encephalopathy, the most common PRES symptoms reported in pediatric cases [5,11]. Seizures usually occur within the first 24 to 48 hours of presentation but can develop later during the disease course. Likewise, status epilepticus may be the presenting symptom [9]. Electroencephalographic monitoring should be performed in every patient with persistent altered levels of consciousness when PRES is suspected [2].

Given the nonspecific neurological symptoms associated with PRES, HTN is frequently a hallmark of the diagnosis and is found in 70% to 80% of patients. However, since HTN can be associated with anxiety or hyperactive sympathetic response triggered by seizures, it can be misinterpreted [15]. Therefore, a high degree of suspicion for PRES is essential in pediatric patients experiencing acute altered mental status or seizures and HTN.

The diagnosis of PRES is established based on clinical features, risk factors, and brain MRI. Since CT scan is widely available for emergent evaluation, it has frequently been used in the acute setting, showing PRES lesions as low-density areas, as in case 2. However, its sensitivity is limited, particularly in the initial phase, as noticed in case 3 [3]. Regarding brain MRI, T2-weighted and FLAIR sequences are the most sensitive, revealing VE as hyperintensity lesions. DWI combined with apparent diffusion coefficient mapping sequences helps differentiate cytotoxic from VE and thus may aid in differentiating PRES from ischemic lesions. Classic imaging patterns usually involve parieto-occipital regions with a bilateral, subcortical, and symmetrical pattern [7]. Our patients also exhibited lesions in other regions, not always symmetrical or confined to subcortical areas. This is consistent with what has been described in some pediatric studies, which are uncovering lesions associated with PRES in locations that differ from previous descriptions [5,11].

Since no specific treatment for PRES is available, supportive care is the mainstay. The management of PRES involves the treatment of the underlying cause or removal/reduction of causative factors, treatment of seizures or status epilepticus, and control of BP. After ruling out cerebral infarction and signs of intracranial HTN, BP should be lowered to near the 99th percentile level for age and sex. Intravenous antihypertensive therapy is preferred to avoid fluctuations in BP. Various drugs can be used, such as nicardipine, labetalol, and sodium nitroprusside, according to physician experience [16]. Other potential therapeutic measures include hydration and correction of fluid overload and electrolyte disturbances [3,7].

PRES is self-limited in most patients. Prompt recognition and approach are essential to achieve reversibility. However, chronic complications can develop, such as neurological impairment or imaging abnormalities, chronic epilepsy, recurrent PRES, or subtle neurological deficits, including development delay and learning disabilities [3]. Some authors recommend a gradual withdrawal of antiepileptic therapy within 3 to 6 months [17], whereas others recommend discontinuation following resolution of PRES, provided that there is adequate control of risk factors and absence of factors that could lower the seizure threshold [18]. The patient described in case 1 presented with EEG abnormal findings at follow-up and required antiepileptic therapy for one year, although no seizures were recorded.

## Conclusions

In conclusion, PRES should always be considered in children with acute neurological impairment presenting with seizures and encephalopathy, especially in the presence of risk factors and HTN. Every physician who provides emergency care to the pediatric population must be familiar with this entity, including its diagnosis and approach, to prevent permanent neurological sequelae. Prompt recognition and management are crucial to prevent complications. Since data are derived mostly from retrospective studies and case series, further research is needed to establish guidelines for its approach.
